# External Validation and Comparison of Two Nomograms Predicting the Probability of Lymph Node Involvement in Patients subjected to Robot-Assisted Radical Prostatectomy and Concomitant Lymph Node Dissection: A Single Tertiary Center Experience in the MRI-Era

**DOI:** 10.3389/fsurg.2022.829515

**Published:** 2022-02-25

**Authors:** Nicola Frego, Marco Paciotti, Nicolò Maria Buffi, Davide Maffei, Roberto Contieri, Pier Paolo Avolio, Vittorio Fasulo, Alessandro Uleri, Massimo Lazzeri, Rodolfo Hurle, Alberto Saita, Giorgio Ferruccio Guazzoni, Paolo Casale, Giovanni Lughezzani

**Affiliations:** ^1^Department of Biomedical Sciences, Humanitas University, Milan, Italy; ^2^Department of Urology, Istituto di Ricovero e Cura a Carattere Scientifico (IRCCS) Humanitas Research Hospital, Milan, Italy

**Keywords:** prostate cancer, lymph node invasion, nomogram, pelvic lymph node dissection, mpMRI

## Abstract

**Introduction:**

To externally validate and directly compare the performance of the Briganti 2012 and Briganti 2019 nomograms as predictors of lymph node invasion (LNI) in a cohort of patients treated with robot-assisted radical prostatectomy (RARP) and extended pelvic lymph node dissection (ePLND).

**Materials and Methods:**

After the exclusion of patients with incomplete biopsy, imaging, or clinical data, 752 patients who underwent RARP and ePLND between December 2014 to August 2021 at our center, were included. Among these patients, 327 (43.5%) had undergone multi-parametric MRI (mpMRI) and mpMRI-targeted biopsy. The preoperative risk of LNI was calculated for all patients using the Briganti 2012 nomogram, while the Briganti 2019 nomogram was used only in patients who had performed mpMRI with the combination of targeted and systematic biopsy. The performances of Briganti 2012 and 2019 models were evaluated using the area under the receiver-operating characteristics curve analysis, calibrations plot, and decision curve analysis.

**Results:**

A median of 13 (IQR 9–18) nodes per patient was removed, and 78 (10.4%) patients had LNI at final pathology. The area under the curves (AUCs) for Briganti 2012 and 2019 were 0.84 and 0.82, respectively. The calibration plots showed a good correlation between the predicted probabilities and the observed proportion of LNI for both models, with a slight tendency to underestimation. The decision curve analysis (DCA) of the two models was similar, with a slightly higher net benefit for Briganti 2012 nomogram. In patients receiving both systematic- and targeted-biopsy, the Briganti 2012 accuracy was 0.85, and no significant difference was found between the AUCs of 2012 and 2019 nomograms (*p* = 0.296). In the sub-cohort of 518 (68.9%) intermediate-risk PCa patients, the Briganti 2012 nomogram outperforms the 2019 model in terms of accuracy (0.82 vs. 0.77), calibration curve, and net benefit at DCA.

**Conclusion:**

The direct comparison of the two nomograms showed that the most updated nomogram, which included MRI and MRI-targeted biopsy data, was not significantly more accurate than the 2012 model in the prediction of LNI, suggesting a negligible role of mpMRI in the current population.

## Introduction

Lymph node invasion represents a key prognostic factor for patients affected by prostate cancer (PCa), being associated with a higher risk of biochemical and disease-recurrence as well as cancer-specific mortality (1, 2). Despite the efforts in developing new imaging techniques for nodal staging, extended pelvic lymph node dissection (ePLND) at the time of radical prostatectomy (RP) still represents the current gold standard for the detection of lymph node invasion (LNI) (3, 4). Although its undiscussed prognostic and staging role, ePLND remains an invasive procedure associated with a non-negligible risk of intra- and post-operative complications, with a controversial therapeutic value (5–8). To limit the number of unnecessary lymphadenectomies, the European Association of Urology (EAU) guidelines recommend performing ePLNDs in patients with a significant risk of LNI according to currently available nomograms (3). Among them, the Briganti 2012 achieved higher accuracy in the prediction of LNI, compared to other nomograms, in several external validation cohorts, being the most applied in Europe (9–12). Most of these tools were developed in the pre-multi parametric magnetic resonance imaging (mpMRI)—era, taking therefore into consideration only clinical and systematic biopsy information. In this context, the novel Briganti 2019 nomogram has been developed in order to include the clinical staging at mpMRI and the Gleason score (GS) at MRI-targeted biopsy, with the aim of exploiting the ability of this diagnostic technique to detect clinically significant (cs) PCa, and thus, better identifying those patients with a greater risk of LNI. Several external validation studies had investigated the accuracy of the Briganti 2019 nomogram, with an AUC ranging between 76 and 80% using a risk threshold of 7% (13–16). However, few studies have directly compared the accuracy of Briganti 2012 and 2019 nomograms, and therefore, the potential added value of mpMRI in nodal staging. Moreover, there is a lack in the literature about the performance of these models in patients with intermediate risk (IR) disease, which is the most heterogeneous category of PCa patients. In this sub-cohort, the indication for ePLND remains indeed greatly controversial. The present study aimed to externally validate and compare the accuracy of Briganti 2012 and 2019 nomograms in a cohort of patients treated with robot-assisted radical prostatectomy (RARP) and anatomically defined ePLND in a tertiary referral hospital.

## Materials and Methods

### Study Design and Data Collection

After institutional review board approval, we retrospectively collected perioperative data of 1,638 consecutive patients treated with RARP and ePLND for clinically localized PCa, between December 2014 and August 2021. Preoperative data included total prostate-specific antigen (PSA) level (ng/ml), clinical T stage (cTstage) based on digital rectal examination (DRE), and the number of positive and total cores, as well as the GS at systematic prostate biopsy. The cTstage was determined according to the cTNM classification 2010. Prostate biopsies were performed according to the EAU guidelines recommended regimen of 12 systematic biopsies. For those patients who underwent mpMRI, we considered the prostate imaging-reported and data system (PI-RADS) score of the main suspected lesion, the maximal target lesion diameter, the presence of extra-capsular extension (ECE) or seminal vesicles invasion (SVI). We included patients with at least one PI-RADS score ≥3 lesion receiving at least two MRI-targeted biopsies for each detected lesion plus at least 10 random biopsies. The primary and secondary GS as well the Gleason group (GG) of biopsy specimens were evaluated according to the ISUP 2014 grading system. All patients were treated with RARP (DaVinci system Xi, Intuitive Surgical, CA, USA) performed by three expert urologic surgeons. No patients received neoadjuvant therapy before surgery. The ePLND template applied involved the removal of nodes overlying the external iliac vessels and internal iliac artery, as well as nodes located within the obturator fossa. Postoperative data included final GG, pathological staging, the total number of resected lymph node, and the number of positive nodes. The preoperative risk of LNI was calculated for all patients using Briganti 2012 nomogram, merging systematic and targeted biopsies for patients who underwent mpMRI and targeted biopsy. The Briganti 2019 nomogram was used only in patients who had performed mpMRI with the combination of targeted and random biopsy. Intermediate Risk (IR) PCa was defined according with D'Amico classification (i.e., PSA 10–20 ng/ml or GS = 7 or cTstage = cT2b).

### Study Outcomes

The primary outcome of the study is to evaluate separately the accuracy and clinical usefulness of both Briganti 2012 and 2019 nomograms. Subsequently, we directly compare the two models in a sub-cohort of patients with mpMRI, mpMRI targeted biopsy, and systematic biopsy. The secondary outcome is to assess and compare the performance of both nomograms in a sub-cohort of patients affected by IR PCa.

### Statistical Analysis

Continuous variables were reported as median and IQR and compared with the Mann-Whitney test. Categorical variables were reported as proportions and compared with the Pearson chi-squared test or Fisher's exact test, as appropriate. External validation of the models followed the TRIPOD recommendations (17). Previously published regression coefficients were used to calculate the individual risk of LNI (14, 18). The performances of Briganti 2012 and 2019 nomograms were evaluated in terms of discrimination, calibration, and clinical usefulness. The accuracy was evaluated using the area under the curve (AUC) of the receiver operating characteristics (ROC) curve. The calibration plot was used to evaluate the concordance between the predicted frequencies (*x*-axis) and the observed frequencies (*y*-axis), assessing in this way the extent of over-and under-estimation of the models. Decision curve analysis (DCA) was used to evaluate the net benefit of the models according to the established cut-off. The same analyses were performed for the entire population and separately for the subgroup of IR PCa. A two-sided *p*-value < 0.05 was taken to indicate statistical significance. Statistical analyses were performed with STATA/SE 17 (StataCorp, College Station, TX, USA).

## Results

Overall, 752 patients presented the available necessary data to calculate the Briganti 2012 nomogram; while the 2019 model was applied in 327 (43.5%) patients with available mpMRI, targeted, and systematic biopsies information. Baseline characteristics of the main patient cohort are presented in Table 1. Overall, 78 patients (10.4%) had LNI on final pathological examination, with a median (IQR) of 13 (9–18) resected nodes. Significant differences were observed between pN0 and pN1 patients in most of the considered characteristics.

**Table 1:** Descriptive characteristics of the main cohort with a comparison between the group with negative and positive lymph nodes.

	**Overall**	**pN0**	**pN1**	***p*-value**
Patients, overall, *n* (%)	752 (100%)	674 (89.6%)	78 (10.4%)	
Median age at surgery, years (IQR)	65 (60–69)	65 (60–69)	64 (60–68)	0.65
Median preoperative PSA, ng/mL (IQR)	7 (5.15–10)	6.77 (5.08–9.4)	10.18 (7.37–16)	<0.0001
**Clinical stage at DRE**, ***n*** **(%)**			
cT1c	540 (71.8%)	502 (74.5%)	38 (48.2%)	<0.0001
cT2	169 (22.5%)	139 (20.6%)	30 (38.5%)	
cT3	43 (5.7%)	33 (4.9%)	10 (13%)	
Patients with positive mpMRI, *n* (*n*%)	327 (100%)	286 (87.5%)	41 (12.5%)	
Median max. index lesion diameter on mpMRI, mm (IQR)	11 (8–14)	10 (8–13)	14 (12–20)	<0.0001
**PI-RADS score for index lesion**, ***n*** **(%)**				<0.0001
3	45 (14%)	44 (15%)	2 (4.2%)	
4	178 (54.5%)	166 (58%)	13 (31.2%)	
5	104 (31.5%)	76 (27%)	26 (64.6%)	
**Clinical stage at MRI**, ***n*** **(%)**				<0.0001
cT2	274 (83.7%)	253 (88.2%)	21 (52%)	
cT3a	36 (10.9%)	28 (9.8%)	8 (18.8%)	
cT3b	17 (5.4%)	5 (1.7%)	12 (29.2%)	
Median number of cores taken, overall, *n* (IQR)	14 (12–15)	14 (12–15)	14 (11–15)	0.8
Median number of pos. cores, overall, *n* (IQR)	5 (3–7)	4 (3–6)	7 (5–10)	<0.0001
**ISUP Grade Group on biopsy, overall**, ***n*** **(%)**				<0.0001
1	201 (26.7%)	196 (29.1%)	5 (5.9%)	
2	305 (40.6%)	291 (43.2%)	13 (17.6%)	
3	125 (16.6%)	102 (15%)	24 (30.6%)	
4	85 (11.3%)	63 (9.4%)	22 (28.2%)	
5	36 (4.8%)	22 (3.3%)	14 (17.7%)	
Median number of pos. systematic cores, *n* (IQR)	3 (2–6)	3 (2–5)	5 (3–8)	<0.0001
Median perecent. (%) of pos. systematic cores with csPCa, *n* (IQR)	21.5%(0–42.5%)	17%(0–41%)	50%(30–92%)	<0.0001
Median number of pos. targeted cores, *n* (IQR)	2 (1–3)	2 (1–3)	3 (2–4)	<0.0001
**ISUP Grade Group on MRI-targeted biopsy**, ***n*** **(%)**				<0.0001
1	56 (17.2%)	58 (20.1%)	1 (2%)	
2	139 (42.5%)	134 (47%)	7 (17.4%)	
3	61 (18.5%)	49 (17.2%)	11 (27.5%)	
4	45 (13.7%)	31 (10.9%)	12 (27.6%)	
5	26 (8%)	14 (4.8%)	10 (25.5%)	
**Pathological Grade Group, overall**, ***n*** **(%)**				<0.0001
1	110 (14.6%)	106 (15.7%)	4 (4.9%)	
2	348 (46.1%)	344 (50.8%)	4 (4.9%)	
3	173 (23%)	149 (22.2%)	24 (30.5%)	
4	67 (9.1%)	48 (7.2%)	19 (25.1%)	
5	54 (7.2%)	27 (4.1%)	27 (34.6%)	
**Pathological Stage, overall**, ***n*** **(%)**				<0.0001
pT2	481 (64%)	469 (69.6%)	12 (15.4%)	
pT3a	161 (21.3%)	141 (20.9%)	20 (25.6%)	
pT3b	110 (14.7%)	64 (9.5%)	46 (59%)	

**Figure 1:**
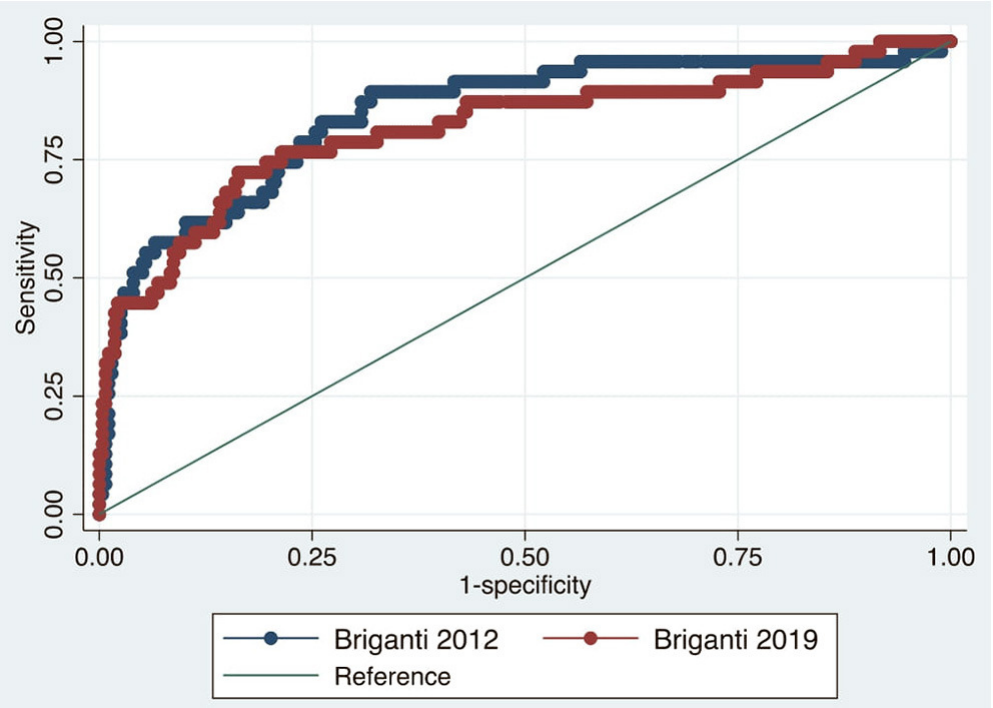
Direct comparison of Briganti 2012 and 2019 ROC curves.

**Figure 2:**
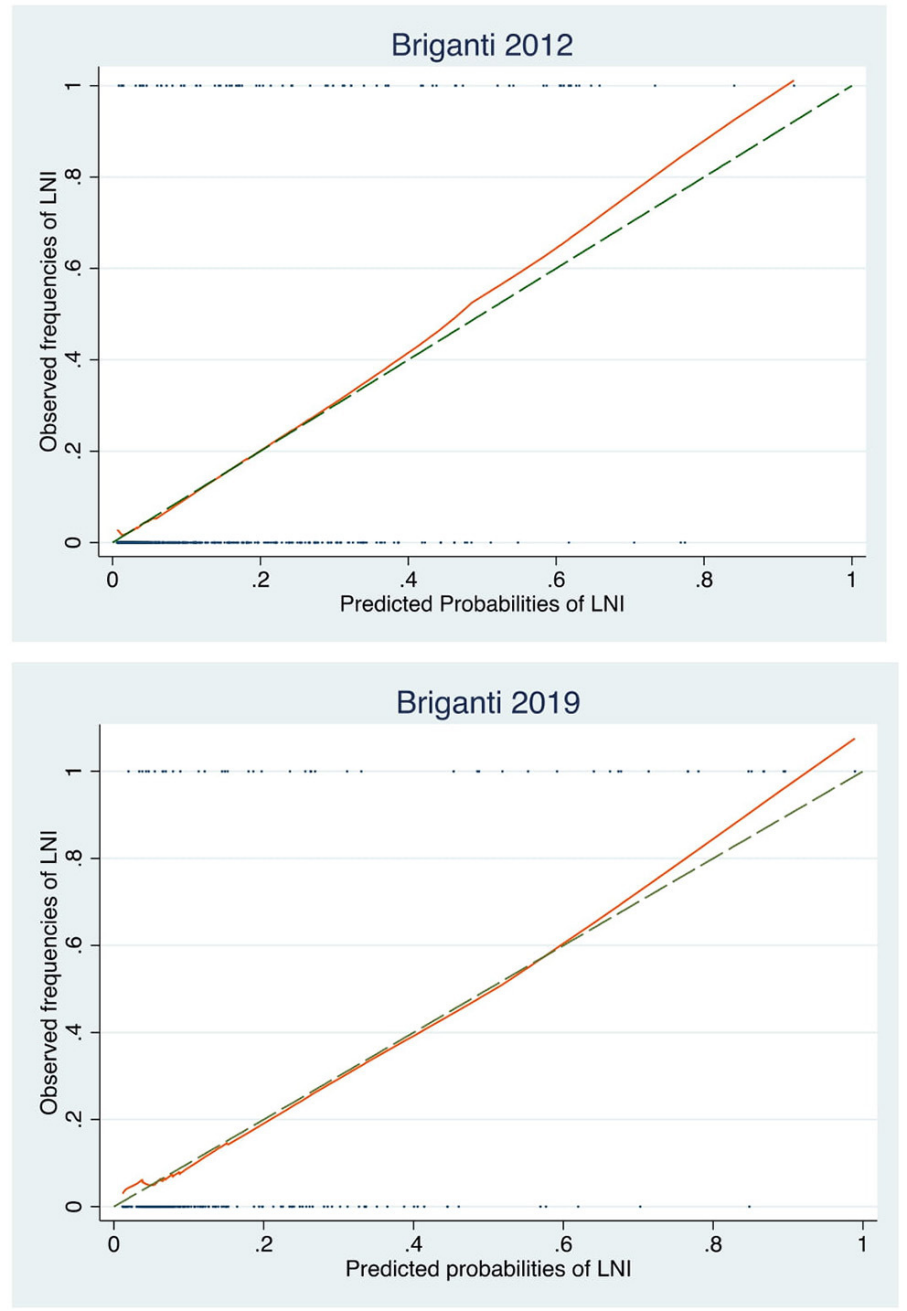
Graphical representation of Briganti 2012 and 2019 calibration plots.

**Figure 3:**
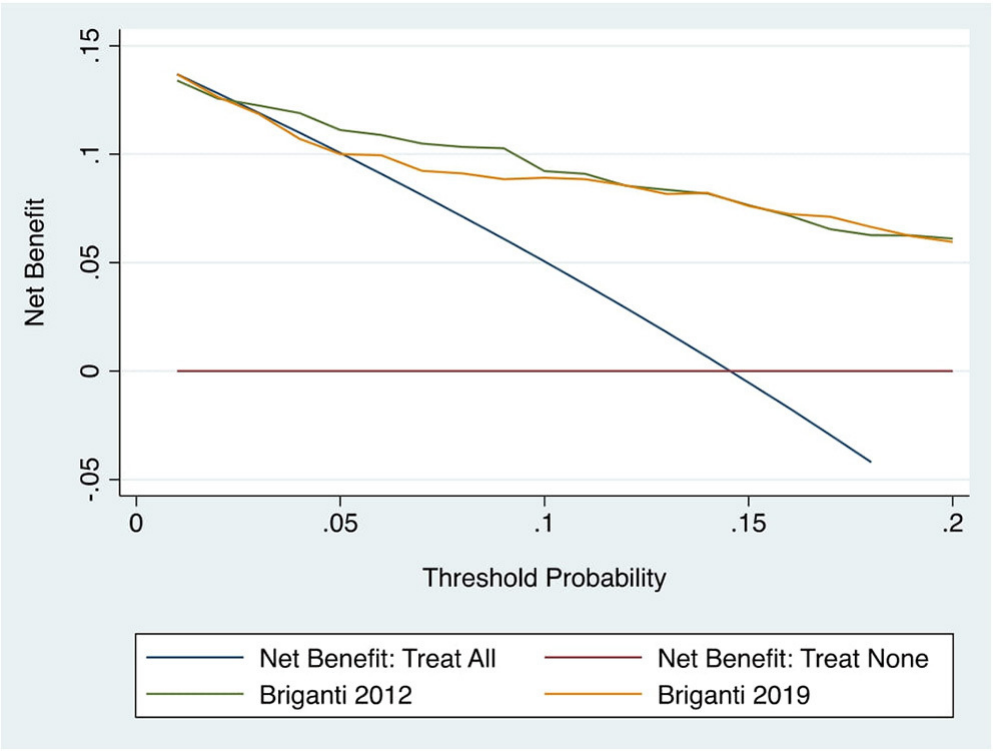
Decision curve analysis of Briagnti 2012 and 2019 nomograms.

The AUCs of Briganti 2012 and Briganti 2019 nomograms were 0.84 and 0.82, respectively. When directly comparing these models in the sub-cohort of 327 patients with MRI data, the AUCs were respectively 0.85 and 0.82, and this difference was not statistically significant (*p* = 0.296) (Figure 1). Graphical representation of calibration plots of both models showed a satisfactory concordance between predicted probabilities and observed frequencies of LNI, with a general slight tendency to underestimation (Figure 2). The DCAs of the two models were similar, with a slightly higher net benefit for Briganti 2012 nomogram in the ability to identify true positive patients and reduce the number of unnecessary PLND, considering threshold probabilities below 20% (Figure 3).

Table 2 shows the proportion of missing LNI according to the guidelines suggested cut-off of 5% and 7% for Briganti 2012 and 2019, respectively. Briganti 2012 allowed to avoid 56.2% of ePLNDs missing 2.4% of patients with LNI, while using Briganti 2019 nomogram would result in 54.1% of ePLNDs avoided and 5.1% missed patients with LNI.

**Table 2:** Results of Briganti 2012 and 2019 application.

**(A) Total population**		
	**Briganti 2012 <5%**	**Briganti 2012 ≥5%**	**Total**
pN0	413 (97.6%)	261 (79.3%)	674 (89.6%)
pN1	10 (2.4%)	68 (20.7%)	78 (10.4%)
Total	423 (56.2%)	329 (43.8%)	**752 (100%)**
**(B) mpMRI population**		
	**Briganti 2012 <5%**	**Briganti 2012 ≥5%**	**Total**
pN0	153 (97.4%)	132 (40.5%)	285 (87.1%)
pN1	4 (2.6%)	38 (11.7%)	42 (12.8%)
Total	157 (48%)	170 (52%)	**327 (100%)**
	**Briganti 2019 <7%**	**Briganti 2019 ≥7%**	**Total**
pN0	168 (94.9%)	112 (74.7%)	280 (85.6%)
pN1	9 (5.1%)	38 (10.7%)	47 (14.4%)
Total	177 (54.1%)	150 (45.9%)	**327 (100%)**

*(A) refers to the total population (752 patients); (B) refers to the mpMRI population (327 patients)*.

Among the entire population, 518 (68.9%) patients harbored intermediate-risk PCa according to D'amico criteria. In this sub-cohort, the Briganti 2012 AUC was 0.82, while in the 237 (45.7%) intermediate-risk patients with positive mpMRI, positive targeted, and systematic biopsies the Briganti 2019 AUC was 0.77. Comparing the accuracy of the two models in these 237 patients the AUCs were 0.85 and 0.77, respectively for the 2012 and 2019 Briganti nomogram, and this difference was statistically significant (*p* = 0.0413). Calibration plots were similar for both the models, while the DCA showed a better net benefit for the Briganti 2012 compared to Briganti 2019 nomogram. Finally, considering a cut-off of 5% for Briganti 2012, 269 out of 518 (51.9%) ePLNDs might have been avoided among IR PCa patients, missing 8 (3%) LNI; using a cut-off of 7% for Briganti 2019, 114 out of 237 (48.1%) IR patients would have been spared from a useless ePLNDs, missing 5 (4.2%) cases of LNI.

## Discussion

The importance of performing an accurate nodal staging in patients with PCa is essential for treatment planning and subsequent follow-up. According to current guidelines, the decision to perform nodal dissection should be guided by the pre-operative probability of LNI. The purpose of the current study is to externally validate and directly compare the performance of the Briganti 2012 nomogram, which is the most applied model in Europe, and the recently introduced Briganti 2019, which in addition to the first one, includes mpMRI and mpMRI-targeted biopsy data. Our results showed a similar accuracy to discern between patients with and without LNI, with non-statistically different AUCs for Briganti 2012 and 2019. Similar results were observed for calibration analysis and clinical net-benefit at DCAs, with a slight advantage of Briganti 2012 over the 2019 model. These results were in line with those observed in previous external validation studies. Oderda et al. performed a multi-institutional external validation of several nomograms for the prediction of LNI, including both Briganti 2012 and 2019 nomograms. The results of the direct comparison in 444 patients, showed how the 2012 model outperformed the 2019 nomogram in terms of accuracy (AUC: 0.79 vs. 0.76, respectively) and net-benefit, although without a statistically significant difference (16). Diamand et al. found the same AUC of 0.8 for both Briganti 2012 and 2019, but with a better net benefit for the 2019 model (15). On the other hand, Gandaglia et al. in a multi-institutional validation cohort comparing the performance of Briganti 2019 nomogram with those of Briganti 2012, 2017 and Memorial Sloan Kettering Cancer Center models, found a higher AUC for Briganti 2019 (79 vs. 75 vs. 65 vs. 74%), a better calibration and the highest net-benefit (13). These findings show how the role of mpMRI and mpMRI-targeted biopsy in PCa staging is still debated and not well-defined, especially with regards to nodal staging. It is well-established that the sensitivity and specificity of mpMRI in the direct detection of metastatic nodes, based on morphological characteristics, are not sufficient (19, 20); however, several studies have found that the presence of ECE and SVI at mpMRI are independent predictors of adverse pathological features such as Extra-Prostatic Disease (EPE), GG upgrading and LNI (21–23). Multiparametric-MRI has been shown to have higher accuracy than clinical and biopsy quantitative histology features in predicting EPE (24–27). Despite this, mpMRI remains characterized by a suboptimal capability to identify the presence of ECE and SVI with high specificity but a poor sensitivity, as shown by the statistically significant difference between clinical MRI stage and pathological stage at RP (*p* < 0.0001) observed in our study (24, 28–30). Actually, mpMRI is not considered a reliable tool for local staging of PCa, and its suboptimal rate of ECE and SVI detection could negatively affect the ability of the 2019 nomogram to predict LNI. This lack of performance improvement could also be explained by other clinical and technical characteristics. First, the high variability of mpMRI interpretation among different readers and a lack of standardization of EPE criteria could impact the reproducibility and the accuracy of the technique (31). Second, MRI-targeted biopsy is a highly operator-dependent procedure, with wide variability in terms of results according to operator experience (32). Finally, in our study, we included mpMRI conducted with both 1.5 T and 3 T magnetic fields, where 1.5 T procedures were certainly less precise in identifying extra-prostatic diseases (33).

We also analyzed the performance of both Briganti 2012 and 2019 nomograms in a sub-cohort of 518 IR PCa patients. Intermediate risk represents the most frequent but also the most heterogeneous PCa category, with a reported rate of LNI ranging from 3.7% to 20.1% (34). In this population, the role of ePLND is often debated and therefore the nomograms play a major role in deciding whether to perform ePLND. In contrast with the results in the general population, in the IR group, the Briganti 2012 nomogram significantly outperformed the Briganti 2019 in terms of accuracy (0.85 vs. 0.77). Moreover, the older nomogram presented a better calibration and a higher net benefit compared to the newer one. As previously discussed, also in the IR PCa patients we found fewer cases of ECE and SVI identified on mpMRI compared to those found on final pathological examination, which might affect the ability of the 2019 model to identify the LNI.

Overall, our work showed that, in a real-life setting, mpMRI and MRI-targeted biopsy provide a limited additional value in improving the accuracy of clinical predictors of LNI. In this context, possible future developments could come from the widespread adoption of PSMA PET/CT. The diagnostic accuracy of PSMA PET/CT was found to be significantly higher than that of conventional imaging techniques in the detection of nodal metastasis in intermediate-high risk PCa, although not such as to replace ePLND (35–37). On the contrary, its role in the local staging remains controversial with several limitations and has not been shown to be superior to mpMRI (38–40). The first study that incorporated the PSMA PET/TC findings in the commonly used nomograms for the prediction of LNI showed an improvement in all models' performance (41). Therefore, it is likely that in the future ePLND will be guided by nomograms integrating both mpMRI and PSMA PET/CT data.

This study is not devoid of limitations. First, it was not possible to perform a direct comparison of both nomograms in the whole population, due to the lack of MRI data in a good proportion of patients. This limited the power of our analysis and may have affected the significance of our results. Second, the study included patients receiving MRI both at our institution and externally. Lack of radiological review of external cases may have negatively affected the performance of MRI in the current population. Third, the lack of central pathological examination of biopsy specimens may have also significantly affected our findings. Finally, the monocentric retrospective nature of the study could limit the generalizability of our results.

## Conclusion

Our study shows that both Briganti 2012 and 2019 nomograms have similar accuracy in predicting the risk of LNI in PCa patients. However, the Briganti 2012 model showed a slightly better accuracy, calibration, and net benefit than the 2019 nomogram, especially in the sub-group of IR PCa patients. These results suggest that the role of mpMRI and mpMRI targeted-biopsies in nodal staging is potentially negligible, probably due to the low sensitivity of mpMRI to identify the extracapsular disease and seminal vesicles invasion, which are the best predictors of LNI on mpMRI.

## Data Availability Statement

The raw data supporting the conclusions of this article will be made available by the authors, without undue reservation.

## Ethics Statement

The studies involving human participants were reviewed and approved by humanitas research institute (protocol number: ICH-016). Written informed consent for participation was not required for this study in accordance with the national legislation and the institutional requirements.

## Author Contributions

GL, NF, and NB contributed to conception and design of the study. NF organized the dataset and the collection of data. DM, RC, PA, VF, and AU contributed to the collection of data. NF and MP performed the statistical analysis and write the manuscript. ML, RH, AS, GG, and PC contributed to the manuscript revision. All authors contributed to the article and approved the submitted version.

## Conflict of Interest

The authors declare that the research was conducted in the absence of any commercial or financial relationships that could be construed as a potential conflict of interest.

## Publisher's Note

All claims expressed in this article are solely those of the authors and do not necessarily represent those of their affiliated organizations, or those of the publisher, the editors and the reviewers. Any product that may be evaluated in this article, or claim that may be made by its manufacturer, is not guaranteed or endorsed by the publisher.
